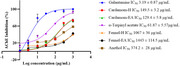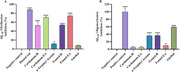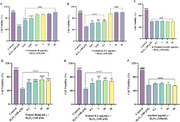# Multivalent Neuroprotective Activity of *Elettaria cardamomum* (Cardamom) and *Foeniculum vulgare* (Fennel) in H_2_O_2_‐Induced Oxidative Stress in SH‐SY5Y Cells and Acellular Assays

**DOI:** 10.1002/alz70859_098500

**Published:** 2025-12-25

**Authors:** Niti Sharma, Seong Soo A An, Himadri Sharma, Hyewon Yang

**Affiliations:** ^1^ Gachon University, South Korea, Seongnam‐si, Gyeonggi‐do Korea, Republic of (South); ^2^ Gachon university, Seongnam Korea, Republic of (South); ^3^ Department of Bionano Technology, Gachon University, Seongnam Korea, Republic of (South)

## Abstract

**Background:**

Elettaria cardamomum (Cardamom) and Foeniculum vulgare (Fennel) are well‐known spices and are also used as natural mouth fresheners. This study was performed to evaluate their neuroprotective ability based on certain acellular and cellular assays.

**Methods:**

Hexane and ethyl acetate extracts were prepared using cardamom and fennel seeds. GC/MS was performed for the identification of important bioactive compounds. Cell‐based assays were performed using SH‐SY5Y cells. Hydrogen peroxide was used for the induction of oxidative stress, and evaluation was done based on neuroprotection, reduced reactive oxygen species, and restoration of mitochondrial membrane potential (MMP). Additionally, anti‐Aβ fibrillization/oligomerization activities were also analyzed along with anti‐acetylcholinesterase activity.

**Result:**

α‐Terpinyl acetate and anethol were identified as major phytocompounds in cardamom and fennel, respectively. Cardamom extracts and α‐terpinyl acetate were more potent acetylcholinesterase (AChE) inhibitors than fennel extracts and anethol [IC_50_ cardamom extracts, 130–150 μg/mL; α‐terpinyl acetate, 61.87 μg/mL; anethol, 374.2 μg/mL; fennel extracts, >1 mg/mL] and showed mixed‐type inhibition. Only the extracts displayed potent anti‐Aβ fibrilization activity (>50%). Anethol showed potent anti‐Aβ oligomerization activity (>50%), followed by α‐terpinyl acetate and fennel‐H (∼36%). The neuroprotective potential of the spice extracts/phytochemicals was evaluated in SH‐SY5Y cells by using H_2_O_2_‐induced oxidative stress. Cardamom‐EA displayed the best neuroprotection (0.01 to 30 μg/mL). No neuroprotection was observed by α‐terpinyl acetate and anethol. Cardamom extracts and fennel‐H restored the normal reactive oxygen species (ROS) levels at 30 µg/mL and 1 µg/mL, respectively.

**Conclusion:**

Overall, the extracts provided better neuroprotection than the pure compounds in cellular assays and displayed strong anti‐Aβ fibrilization activity.